# Motion-robust high-resolution 3D diffusion-weighted vessel wall imaging at 3T

**DOI:** 10.1186/1532-429X-15-S1-W26

**Published:** 2013-01-30

**Authors:** Y Xie, Z Fan, D Li

**Affiliations:** 1 Cedars-Sinai Medical Center, Los Angeles, CA, USA; 2 University of California, Los Angeles, Los Angeles, CA, USA

## Background

Atherosclerotic plaque evaluation has gone beyond simply determining stenosis percentage to characterizing specific plaque components, such as necrotic lipid core and intraplque hemorrhage, in order to detect high-risk subclinical lesions. Diffusion-weighted imaging (DWI) provides excellent contrast-to-noise for identifying plaque components ex vivo [1] and is shown feasible for in vivo imaging of carotid arterial wall with good correlation to histology [2]. However, current 2D methods based on echo-planar imaging (EPI) have limited resolution, especially in slice-direction, leading to partial volume effects and difficult diffusion quantification. Generally, the use of 3D acquisition improves SNR and resolution at the expense of higher motion sensitivity. The purpose of this work is to develop a high resolution 3D DWI for vessel wall robust to motion.

## Methods

**Figure 1:**
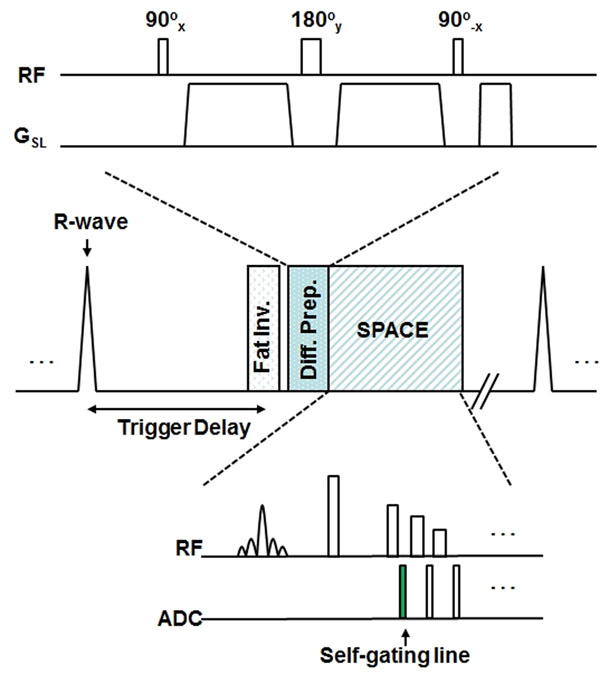
Sequence diagram of diffusion-prepared 3D TSE with motion self-gating.

A 3D variable flip angle TSE sequence (SPACE) is proceeded by a spin echo diffusion weighted preparation with BIR4 refocusing pulse. (Figure 1) An additional self-gating (SG) readout is enabled for real-time motion gating. When bulk motion is detected, current lines will be reacquired in the next repetition as described in [3]. Two healthy volunteers with IRB approval were scanned on a 3T clinical scanner (MAGNETOM Verio, Siemens) with the following parameters: TE/TR = 22 ms/two heartbeats; 3D transverse slab with left-right readout; FOV = 135×135×26 mm2, matrix = 192×192×18, slice thickness = 1.4 (0.7 interpolated) mm, yielding isotropic resolution = 0.7 mm3; ETL = 39; echo spacing = 4.5 ms; bandwidth = 668 Hz/pixel; signal average = 1.5; b = 10 and 330 s/mm2 along the slice direction; acquisition time = 3-4 min for each b-value depending on subject heart rate.

## Results

**Figure 2:**
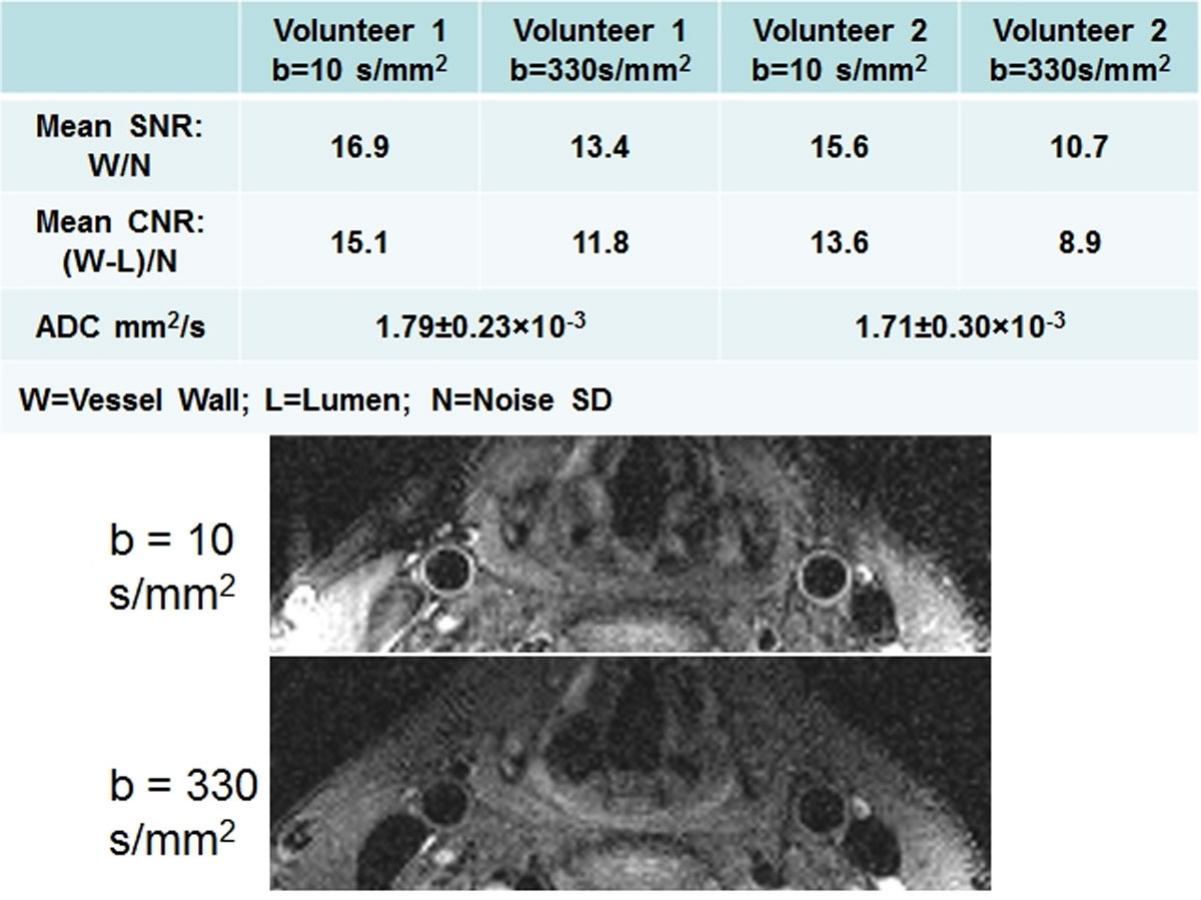
Top panel: mean relative SNR, CNR and vessel wall ADC values for two normal volunteers; Bottom panel: 3D TSE DWI of normal carotid vessel wall at two b values.

Region-of-interest analysis (vessel wall, lumen, remote area) was performed for both volunteers for the calculation of relative SNR, CNR (vessel wall to lumen), and wall ADC values. (Figure 2) At both b-values, images showed clean delineation of vessel wall with SNR in the range of 10 to 17, as well as effective blood suppression throughout the slices with CNR in the range of 9 to 15. Normal vessel wall ADC values are 1.79±0.23×10-3 and 1.71±0.30×10-3 mm2/s for volunteer 1 and 2, respectively.

## Conclusions

A novel DWI 3D sequence is implemented for vessel wall imaging with high resolution (0.7×0.7×1.4 mm3) and robustness to motion. Preliminary studies from healthy volunteers at 3T achieved good quality carotid vessel wall images with reasonably high SNR and CNR. ADC values are consistent with previous in vivo [2] and ex vivo [4] studies. Blood suppression is satisfactory with inherent TSE black-blood effect combined with extra blood dephasing by the preparation. Real-time motion self-gating prevents motion artifacts. Further study of normal and diseased subjects is warranted. Coronary vessel wall application is under development.
